# Histone Deacetylases and NF-kB Signaling Coordinate Expression of CX3CL1 in Epithelial Cells in Response to Microbial Challenge by Suppressing miR-424 and miR-503

**DOI:** 10.1371/journal.pone.0065153

**Published:** 2013-05-28

**Authors:** Rui Zhou, Ai-Yu Gong, Dongqing Chen, Ryan E. Miller, Alex N. Eischeid, Xian-Ming Chen

**Affiliations:** Department of Medical Microbiology and Immunology, Creighton University School of Medicine, Omaha, Nebraska, United States of America; Technion-Israel Institute of Technology Haifa 32000 Israel., Israel

## Abstract

The NF-kB pathway is key to epithelial immune defense and has been implicated in secretion of antimicrobial peptides, release of cytokines/chemokines to mobilize immune effector cells, and activation of adaptive immunity. The expression of many inflammatory genes following infection involves the remodeling of the chromatin structure. We reported here that histone deacetylases (HDACs) and NF-kB signaling coordinate expression of CX3CL1 in epithelial cells following *Cryptosporidium parvum* infection. Upregulation of CX3CL1 was detected in cultured human biliary epithelial cells following infection. Expression of miR-424 and miR-503 was downregulated, and was involved in the induction of CX3CL1 in infected cells. *C. parvum* infection suppressed transcription of the *mir-424-503* gene in a NF-kB- and HDAC-dependent manner. Increased promoter recruitment of NF-kB p50 and HDACs, and decreased promoter H3 acetylation associated with the *mir-424-503* gene were observed in infected cells. Upregulation of CX3CL1 in biliary epithelial cells and increased infiltration of CX3CR1^+^ cells were detected during *C. parvum* infection in vivo. Induction of CX3CL1 and downregulation of miR-424 and miR-503 were also detected in epithelial cells in response to LPS stimulation. The above results indicate that HDACs and NF-kB signaling coordinate epithelial expression of CX3CL1 to promote mucosal antimicrobial defense through suppression of the *mir-424-503* gene.

## Introduction

Epithelial cells along the mucosal surface provide the front line of defense against luminal pathogen infection [1]. The NF-kB pathway is key to epithelial immune defense and has been implicated in secretion of antimicrobial peptides, release of cytokines/chemokines to mobilize immune effector cells, and activation of adaptive immunity [2], [3]. Regulated by histone acetyltransferases and deacetylases (HDACs), histone acetylation is a key epigenetic mechanism controlling chromatin structure, DNA accessibility, and gene expression [4]. Histone acetylation causes the unwinding of the chromatin structure, therefore allowing transcription factor access to promoter sites [4], [5]. HDACs mainly consist of HDAC1, HDAC2, and HDAC type 3 (Sirt1) [4], [5]. As HDACs promote deacetylation, the inhibition of HDAC function increases acetylation of histones and activates gene transcription [4]–[6]. Nevertheless, evidence has accumulated showing that HDAC inhibitors impair cellular immune responses to Toll-like receptor (TLR) agonists and infection [7], [8]. Individual cytokines induced by the NF-kB signaling pathway were reported to be inhibited by HDAC inhibitors, but underlying mechanisms are unclear [7], [8].

*Cryptosporidium parvum*, a zoonotic parasite of the phylum *Apicomplexa*, is the leading cause of waterborne disease outbreaks worldwide [9], [10]. Efforts to develop novel therapeutic strategies have been hampered by the lack of understanding of the pathogenesis of infection. *C. parvum* is classified as a “minimally invasive” mucosal pathogen [10]; and epithelial cells play a central role in activating and orchestrating host immune responses [11]. The invasion of epithelia by *C. parvum* activates NF-kB signaling and triggers host cell defense [12]–[15]. Acquired resistance to cryptosporidial infection requires T-cells with the α/β type T-cell receptor [11]. Infiltration of NK cells, monocytes, lymphocytes (e.g., CD4^+^ and CD8^+^), and dendritic cells has been identified at the site of infection [11]. The chemokine CX3CL1 (also known as fractalkine) is a unique member of the CX3C family, and it binds only to and is the unique ligand of its receptor, CX3CR1 [16]. Unlike other chemokines, CX3CL1 is expressed as a membrane-bound form (95–100 kDa) and can also be shed as a soluble chemotactic form (60–80 kDa). Membrane-bound CX3CL1 is known to function as an adhesion molecule to interact with immune cells that express CX3CR1, including CD4^+^ and CD8^+^ T-cells, NK cells, and monocytes [17], [18]. Nevertheless, little is known mechanistically about the role of CX3CL1 in epithelial defense against pathogens, in particular, *C. parvum*.

miRNAs are small non-coding regulatory miRNAs that identify targets based on complementarity between each miRNA and the 3′-untranslated region (3′UTR) of target mRNAs, resulting in mRNA cleavage and/or translational suppression [19], [20]. We previously demonstrated that activation of TLR4/NF-kB signaling in response to microbial challenge regulates the transcription of a subset of miRNA genes, and that functional manipulation of NF-kB-regulated miRNAs influences epithelial antimicrobial defense [21]–[24]. In this study, we investigated the expression of CX3CL1 in biliary epithelial cells following *C. parvum* infection, its relationship to miRNA- and HDAC-mediated gene regulation, and finally its association with immune cell infiltration to *C. parvum* infection sites in vivo. The data show that induction of CX3CL1 expression in biliary epithelial cells upon microbial challenge involves donwregulation of the miR-424 and miR-503. Histone deacetylases and NF-kB signaling coordinate downregulation of the *mir-424-503* gene and promote mucosal defense through modulating CX3CL1 expression in epithelial cells.

## Materials and Methods

### Ethics statement

This study was carried out in strict accordance with the recommendations in the Guide for the Care and Use of Laboratory Animals of the National Institutes of Health under the Assurance of Compliance Number A3348-01. All animal experiments were done in accordance with procedures (protocol # 868) approved by the Institutional Animal Care and Use Committee of the Creighton University School of Medicine. All surgeries were performed under ketamine and xylazine anesthesia, and all efforts were made to minimize suffering.

### *C. parvum* and infection models

*C. parvum* oocysts of the Iowa strain were purchased from the Bunch Grass Farm (Deary, ID). H69 cells are SV40 transformed normal human biliary epithelial cells originally derived from liver harvested for transplant [25]. An in vitro model of human biliary cryptosporidiosis using H69 cells, as previously reported [15], [21], [22], was employed in these studies. Trichostatin A (TSA, 100 ng/ml), SC514 (100 µM), and EX527 (1 µM) were from Invivogen (San Diego). Lipopolysaccharide (LPS, 1 µg/ml) was from Biomax Technologies (San Diego). These reagents used at above concentrations showed no cytotoxic effects on H69 cells or on *C. parvum* infectivity. All experiments were performed in triplicate.

We adapted a mouse model of biliary and intestinal cryptosporidiosis via gallbladder injection of *C. parvum* originally developed by Verdon [26]. Briefly, *C. parvum* oocysts were treated with 1% sodium hypochlorite on ice for 20 min and directly injected into the gallbladder of C57BL/6J mice (200,000 per 25 µl PBS each animal). Mice were purchased from the Jackson laboratory.

### Western blot and Northern blot

For Western blot, whole cell lysates were obtained with the M-PER Mammalian Protein Extraction Reagent (ThermoScientific, Rockford). Antibodies to CX3CL1 (Santa Cruz) and β-actin (Sigma-Aldrich) were used. Densitometric levels of CX3CL1 were quantified and expressed as their ratio to β-actin. For Northern blot, RNAs were harvested with Trizol reagent. LNA DIG-probes for miR-424 and miR-503 (Exiqon, Vedbaek, Denmark) were hybridized using UltraHyb reagents (Ambion) according to the manufacturer’s instructions, with snRNA RNU6B blotted as a control [21]–[23].

### Quantitative real-time PCR (qRT-PCR)

RNAs were prepared and comparative real-time PCR was performed using the SYBR Green PCR Master Mix (Applied Biosystems) [21]–[23]. RNAs were treated with DNA-free Kit (Ambion) to remove any remaining DNA. The PCR primers were as follows: human CX3CL1 (forward, 5′-CGCAATCATCTTGGAGACGA-3′ and reverse, 5′-GTGCCGCCATTTCGAGTTA-3′); pri-miR-424-503 (the primary transcript of the *mir-424-503* gene) (forward 5′-GGAGTGAAGTGGCCTAGTCATAAG-3′ and reverse 5′-GTATAGCAGCGCCTCACGTT-3′ (reverse); and human GAPDH (forward, 5′-TGCACCACCAACTGCTTAGC-3′ and reverse, 5′-GGCATGGACTGTGGTCATGAG-3′). For real-time PCR analysis of mature miRNAs, PCR primer sets for miR-424 and miR-503 and snRNA RNU6B were obtained from Exiqon (Vedbaek, Denmark). RNAs were reverse-transcribed by using Universal cDNA Synthesis Kit (Exiqon), and real-time PCR was performed in triplicate. The Ct values were analyzed using the comparative Ct (ΔΔCt) method and the amount of target was obtained by normalizing to GAPDH (for mRNA and pri-miRNAs) and snRNA RNU6B (for mature miRNAs) and comparing with the control (non-treated cells) [21]-[23].

### miRCUR LNA array analysis of miRNAs

The Exiqon (Vedbaek, Denmark) miRCURY LNA microRNA arrays and service to process the samples were used [21]–[23]. Briefly, cells were grown to 80% confluence and exposed to *C. parvum* infection for 12 h. A total of 2 µg RNA from each sample was labeled with the Hy^5^ fluorescent label and the reference pool was labeled with Hy^3^ using the miRCURYLNA Array labeling kit (Exiqon). The labeled samples and reference pool were then mixed pair-wise and hybridized to the miRCURY LNA array containing capture probes targeting all human miRNAs listed in the miRBASE version 8.1 (Exiqon). After hybridization, the slides were scanned and quantified signals were normalized by Exiqon using the global Lowess regression algorithm. Normalized Hy^5^/Hy^3^ ratios were used for further analysis as previously reported [21]–[23].

### miR-424 precursor

The miR-424 precursor was obtained from Ambion and used to increase miR-424 expression, as previously reported [21]–[24]. For experiments, cells were grown to 90% confluence and treated with miR-424 precursor (0–30 nM) using the lipofectamine 2000 reagent (Invitrogen). Nonspecific precursor (Ambion) was used as the control (precursor-Ctrl).

### Luciferase reporter constructs and luciferase assay

Complementary 35 bp DNA oligonucleotides containing the putative miR-424 and miR-503 target site within the 3′ untranslated region (3′UTR) of human CX3CL1 were synthesized with flanking *SpeI* and *HindIII* restriction enzyme digestion sites (Sense, 5′-CTAGTGGCCTCTGCACTCCCCTGCTGGGT GTGGCGCAGC-3′; antisense, 5′-AGCTGCTGCGCCACACCCAGCAGGGGAGTGCAGA GGCCA-3′) and cloned into the multiple cloning site of the pMIR-REPORT Luciferase vector (Ambion). A pMIR-REPORT Luciferase construct containing mutant 3′UTR (Sense, 5′-CTAGTGGCCTCTGCACTCCCCACGTGGGTGTGGCGCAGC-3′; antisense, 5′-AGCTGCTGCGCCACACCCACGTGGGGAGTGCAGAGGCCA) was also generated and used as the control. We then transfected cultured cells with each reporter construct and β-gal (as the internal control), as well as an miR-424 precursor. Luciferase activity was measured and normalized to the control β-gal level as previously reported [24].

### Chromatin immunoprecipitation (ChIP) and co-immunoprecipitation (Co-IP)

ChIP analysis was performed with the ChIP Assay Kit (Upstate Biotechnologies) in accordance with the manufacturer’s instructions. In brief, 10^6^ H69 cells were exposed to *C. parvum* infection for 8 h. The chromatin fraction was immunoprecipitated overnight at 4°C using antibodies to p65 (Upstate Biotechnologies), p50 (Santa Cruz), HDAC1 (Santa Cruz), HDAC2 (Santa Cruz), Sirt1 (Santa Cruz), C/EBPβ (Abcam). A histone H3ac pan-acetyl (H3ac) polyclonal antibody (Active Motif) was used for ChIP. qRT-PCR amplification was performed in a total volume of 20 µl with specific primers. ChIP primers for the NF-kB binding site regions of the *mir-424-503* gene promoter were: site A (forward, 5′-CTCTCGGACGCGGCGAAACAG-3′ and reverse 5′-CCACGTTACAGTCGGGAAAG-3′) and site B (forward, 5′-GTTTAACAAATGAGTGCGGC-3′ and reverse 5′-TTCGGGAGAGACAATGTGA-3′). For Co-IP, antibodies to p50, C/EBPβ, or Sirt1 were used; and immunoprecipitations and immunoblotting were performed, as previously reported [27].

### Immunohistochemistry

*C. parvum* infection in the intrahepatic bile ducts in the animals was observed one week post-injection. Five animals from each group were sacrificed and liver tissues obtained for immunohistochemistry, as previously reported [22], [26], [28]. Antibodies to CX3CL1 and CX3CR1 (Santa Cruz) were used.

## Results

### Infection induces expression of CX3CL1 in biliary epithelial cells in a HDAC- and Dicer-dependent manner

**Figure 1:**
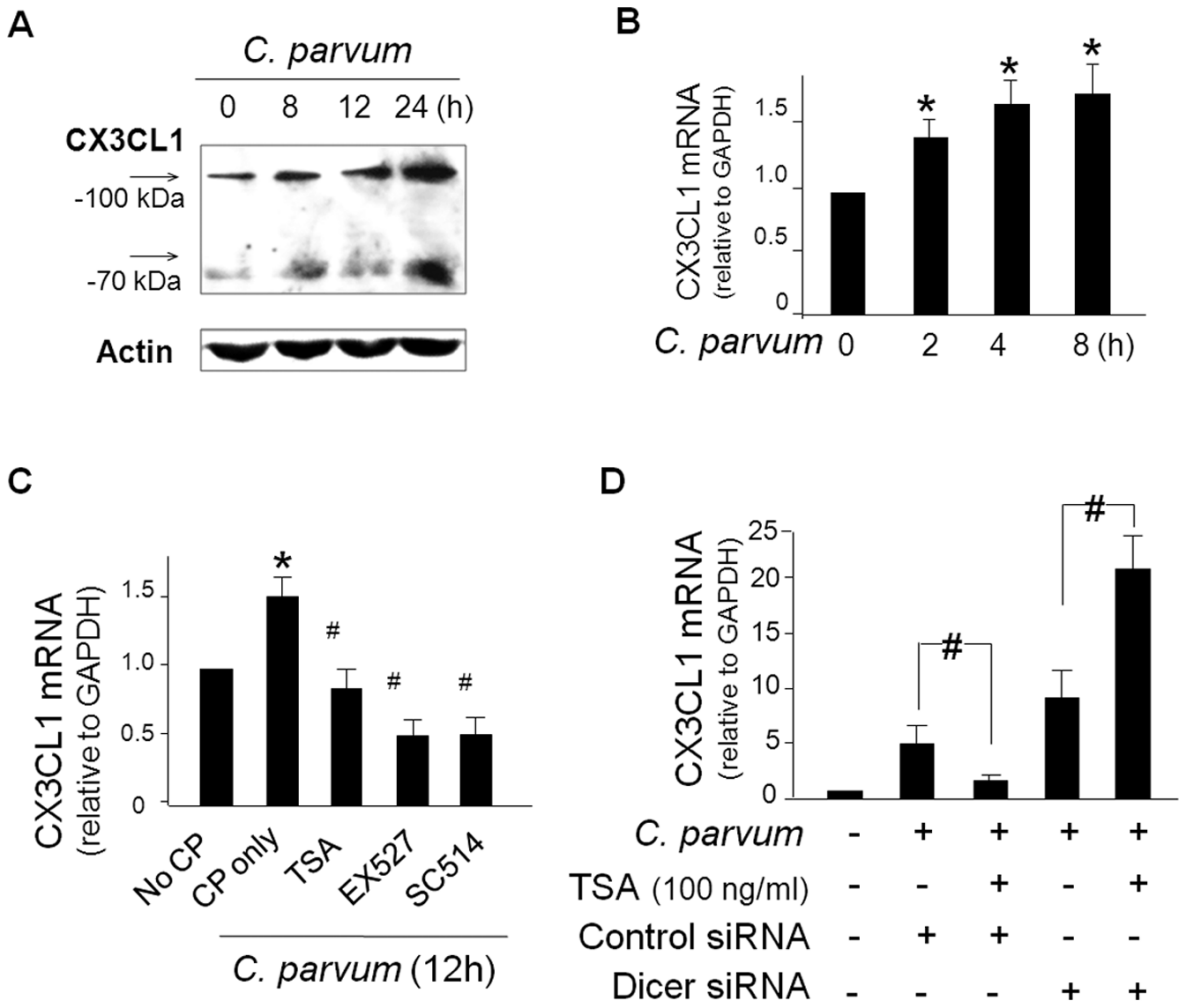
*C. parvum* infection increases expression of CX3CL1 in biliary epithelial cells in a HDAC- and Dicer-dependent manner. **A** and **B**, H69 cells were exposed to *C. parvum* for up to 24h, followed by Western blot **(A)** and qRT-PCR **(B)** analysis for CX3CL1. Representative Western blots were shown and β-actin was blotted as the protein loading control. GAPDH mRNA was used to normalize the CX3CL1 mRNA levels. **C**, CX3CL1 mRNA levels in H69 cells following *C. parvum* infection for 12h in the presence of in the HDAC inhibitors, TSA and EX527, and NF-kB inhibitor SC514, as analyzed by qRT-PCR. Treatment of cells with TSA, EX527, or SC514 attenuated *C. parvum*-induced upregulation of CX3CL1. **D**, Knockdown of Dicer blocked the inhibitory effects of TSA on *C. parvum*-induced CX3CL1 expression. Cells were treated with Dicer siRNA and then exposed to *C. parvum* for 12h in the absence or presence of TSA, followed by qRT-PCR analysis for CX3CL1. Data in B to D are averages of three independent experiments. *, p<0.05 ANOVA vs. the non-infected control (in **A–D**); ^#^, p<0.05 ANOVA vs. *C. parvum* infected cells (in **C**) or non-TSA-treated cells (in **D**).

When H69 cells were exposed to *C. parvum* for infection for up to 24 h, a time-dependent increase in CX3CL1 both at the protein and message levels was detected (Fig. 1*A and 1B*). Western blot reveals double bands for CX3CL1 at 100 kDa and 70 kDa, as previous studies indicated [16]. It appears that both the 100 kDa and 70 kDa bands showed an increase in infected cells. Inhibition of NF-kB signaling by an IKK2 inhibitor, SC514 [29], blocked *C. parvum*-induced expression of CX3CL1 (Fig. 1*C*).

HDACs mediate histone deacetylation and thus suppress gene transcription [4], [5]. We measured CX3CL1 mRNA levels in cells following infection in the presence of HDAC inhibitors, TSA (for HDAC1/2) and EX527 (for HDAC3 type Sirt1) [30], [31]. Unexpectedly, we detected a significant inhibition of *C. parvum*-induced CX3CL1 mRNA expression in infected cells in the presence of HDAC inhibitors (Fig. 1*C*), suggesting an indirect mechanism of HDACs on CX3CL1 expression.

We used an siRNA to knock down Dicer to prevent maturation of miRNAs in cells and then exposed them to *C. parvum* for infection in the presence of TSA. Knockdown of Dicer attenuated the inhibitory effects of TSA on *C. parvum*-induced CX3CL1 expression (Fig. 1*D*). When cells were exposed to LPS, an increase of CX3CL1 expression (at both message and protein levels) was detected (Fig. S1). Inhibition of NF-kB signaling by SC-514 and HDACs by TSA and Sirt1 attenuated LPS-induced CX3CL1 expression (Fig. S2). Taken together, the above data suggest that NF-kB-regulated CX3CL1 expression in epithelial cells upon microbial challenge involves HDAC- and miRNA-mediated gene regulation mechanisms.

### Expression of miR-424 and miR-503 is downregulated, and is involved in the induction of CX3CL1 in epithelial cells following infection

**Figure 2:**
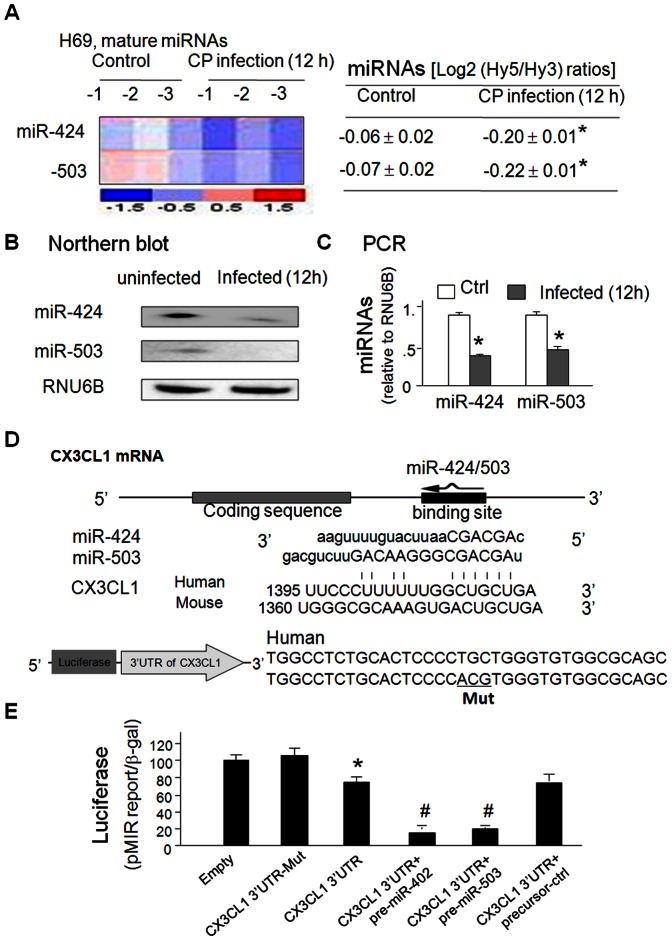
Downregulation of miR-424 and -503 in epithelial cells following *C. parvum* infection, and targeting of CX3CL1 3UTR by miR-424 and miR-503. **A–C**, H69 cells were exposed to *C. parvum* for up to 8h, followed by microarray **(A)**, Northern blot **(B)** and qRT-PCR **(C)** analysis for miR-424 and -503. Levels of miR-424 and -503 by microarray are shown in a heat-map and presented as the log_2_ (Hy5/Hy3) ratios. **D**, The schematic of CX3CL3 mRNA showed a potential binding site in its 3’UTR for miR-424 and -503 in humans and mice. The CX3CL1 3'UTR sequence covering the potential binding site for miR-424 and -503 was inserted into the pMIR-REPORT luciferase plasmid. A control plasmid with the mutant 3'UTR sequence was also generated for control. **E**, Luciferase activity analysis with the pMIR-REPORT luciferase plasmid covering the potential binding site in the CX3CL1 3'UTR in epithelial cells. H69 cells were transfected with the pMIR-REPORT luciferase constructs and treated with the precursors to miR-424, -503, or non-specific oligo control, for 24h, followed by luciferase analysis. Data in **B**, **C** and **E** are averages of three independent experiments. Mut  =  mutant; *, p<0.05 ANOVA vs. the empty vector controls; #, p<0.05 ANOVA vs. cells transfected with CX3CL1 3'UTR only.

H69 cells were exposed to *C. parvum* for up to 12 h and microarray analysis revealed a significant decrease in miR-424 and miR-503 levels in their mature forms (Fig. 2*A*). Downregulation of miR-424 and miR-503 in infected cells was further confirmed by Northern blot (Fig. 2*B*) and qRT-PCR (Fig. 2*C*). Downregulation of miR-424 and miR-503 was also detected in H69 cells following LPS stimulation (Fig. S3). All microarray data were deposited at ArrayExpress (accession number: E-MEXP-2050 and E-MEXP-2052), as described in our previous studies [21], [22].

Both miR-424 and -503 display complementarity to the same region of CX3CL1 3′UTR (Fig. 2*D*). To test the potential targeting of CX3CL1 3′UTR by miR-424 and -503, we inserted the CX3CL1 3'UTR sequence covering the potential binding site for miR-424 and miR-503 into the pMIR-REPORT luciferase plasmid. A control plasmid with the mutant 3'UTR sequence was also generated for control (Fig. 2*D*). H69 cells were transfected with the pMIR-REPORT luciferase constructs and treated with the miR-424 precursor, or non-specific precursor control, for 24 h, followed by luciferase analysis. We detected a significant decrease of pMIR-REPORT- CX3CL1 3′UTR-associated luciferase activity in transfected cells. Overexpression of miR-424 and miR-503 by transfection of their precursors further decreased the luciferase activity (Fig. 2*E*), suggesting targeting of CX3CL1 3′UTR by miR-424 and miR-503.

**Figure 3:**
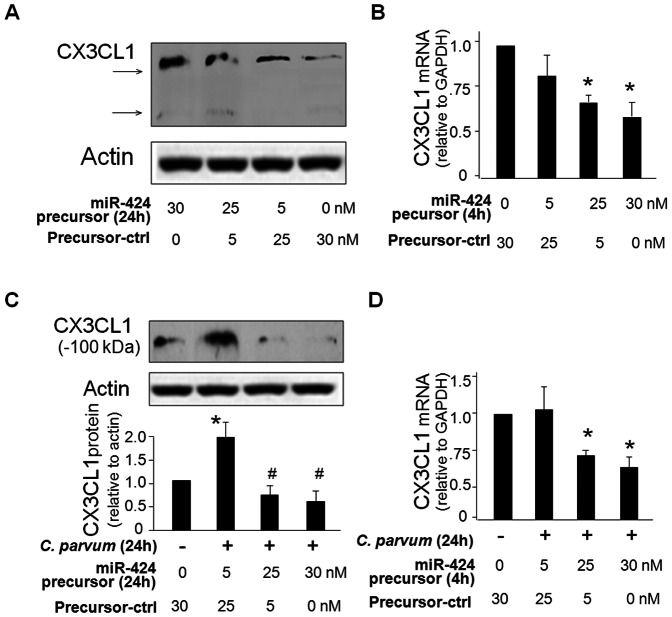
miR-424 precursor decreases CX3CL1 expression at both protein and message levels, and attenuates *C. parvum*-induced upregulation of CX3CL1 in biliary epithelial cells. **A** and **B**, miR-424 precursor decreased CX3CL1 expression at both protein **(A)** and message **(B)** levels in H69 cells. Treatment of H69 cells with miR-424 precursor resulted in a significant decrease in CX3CL1 protein content (24h) and its message level (4h). Representative Western blots were shown and β-actin was blotted as the protein loading control. GAPDH mRNA was used to normalize the CX3CL1 mRNA levels. **C** and **D**, Treatment of cells with miR-424 precursor attenuated CX3CL1 upregulation induced by *C. parvum* infection. H69 cells were exposed to *C. parvum* infection in the presence of miR-424 precursor, followed by Western blot (24h, **C**) and qRT-PCR (4h, **D**) analysis for CX3CL1. Representative Western blots were shown and densitometric ratio to β-actin was presented. Data in **B** to **D** are averages of three independent experiments. *, p<0.05 ANOVA vs. the non-treated cells.

To test whether miR-424 is directly relevant to CX3CL1 expression, we treated H69 cells with miR-424 precursor for 24 h and then measured CX3CL1 protein content. Transfection of cells with the miR-424 precursor caused a dose-dependent decrease in CX3CL1 protein level (Fig. 3*A*). A significant decrease in CX3CL1 mRNA levels was found between the control cells and cells treated with miR-424 precursor for 4 h (Fig. 3*B*), suggesting that miR-424 may promote CX3CL1 mRNA degradation. When H69 cells were transfected with various doses of miR-424 precursor for 24 h and then exposed to *C. parvum* for 24 h, no significant increase in CX3CL1 protein level was detected in the infected cells (Fig. 3*C*). Accordingly, a decrease of CX3CL1 mRNA levels was observed in infected cells after treated with the miR-424 precursor (Fig. 3*D*). Coupled with the downregulation of miR-424 and miR-503 in cells following *C. parvum* infection, the above data suggested that the relief of miR-424- and miR-503-mediated post-transcriptional repression was required for *C. parvum*-induced suppression of CX3CL1 in host epithelial cells.

### *C. parvum* infection suppresses transcription of the pri-mir-424-503 in biliary epithelial cells in a NF-κB- and HDAC-dependent manner

**Figure 4:**
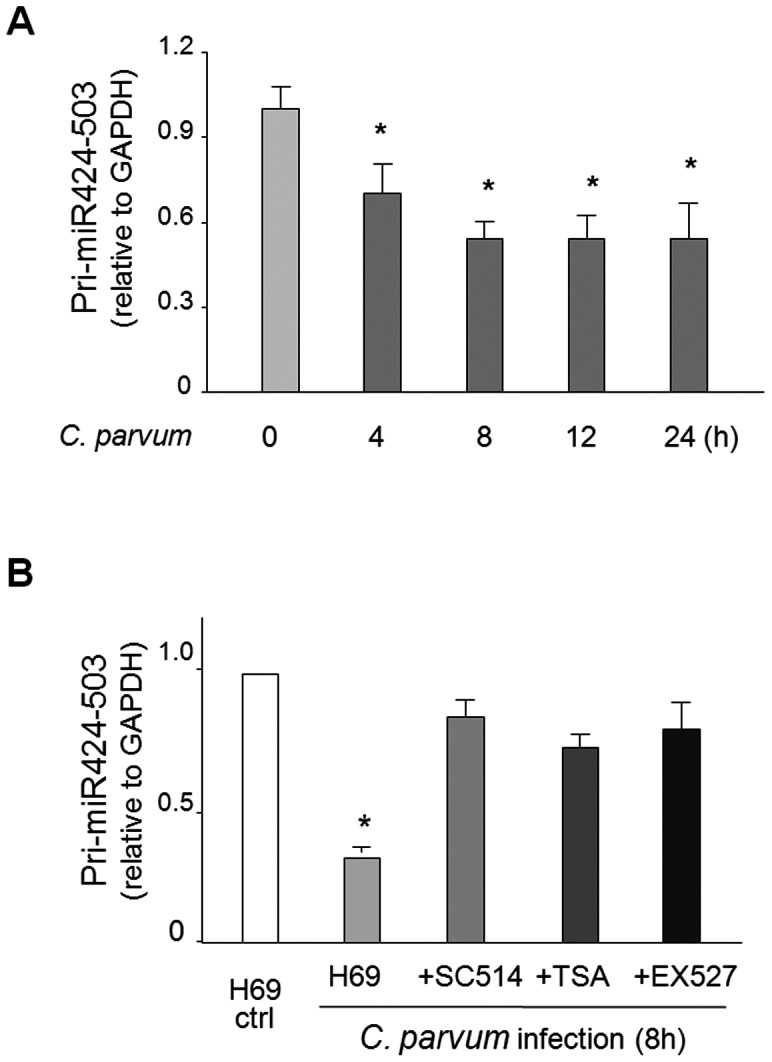
Inhibitors to NF-kB and HDACs attenuate *C. parvum*-induced suppression of pri-miR-424-503 in epithelial cells. **A,** *C. parvum* infection decreased expression of pri-miR-424-503 (primary transcript of the *mir-424-503* gene) in H69 cells in a time-dependent manner, as assessed by qRT-PCT. **B,** Inhibition of NF-kB (by SC-514) and HDACs (by TSA or EX527) abolished *C. parvum*-induced repression of pri-miR-424-503 in H69 cells. Data are averages of three independent experiments. *, p<0.05 ANOVA vs. the non-infected cells.

The *mir-424-503* gene locus at chromosome X codes the mature form of both miR-424 and miR-503 [32]. We detected a significant decrease in the level of pri-miR-424-503 (primary transcript of the *mir-424-503* gene) in cells following *C. parvum* infection for 8 h (Fig. 4*A and 4B*). Importantly, *C. parvum*-induced suppression of pri-miR-424-503 was attenuated by treatment of cells with the inhibitors to NF-kB and HDACs (Fig. 4*B*), suggesting that suppression of the *mir-424-503* gene in cells following *C. parvum* infection involves NF-kB- and HDAC-mediated regulatory mechanisms.

### *C. parvum* infection increases promoter recruitment of NF-kB p50 and HDACs, and decreases H3 acetylation associated with the NF-kB binding promoter region of the mir-424-503 gene

**Figure 5:**
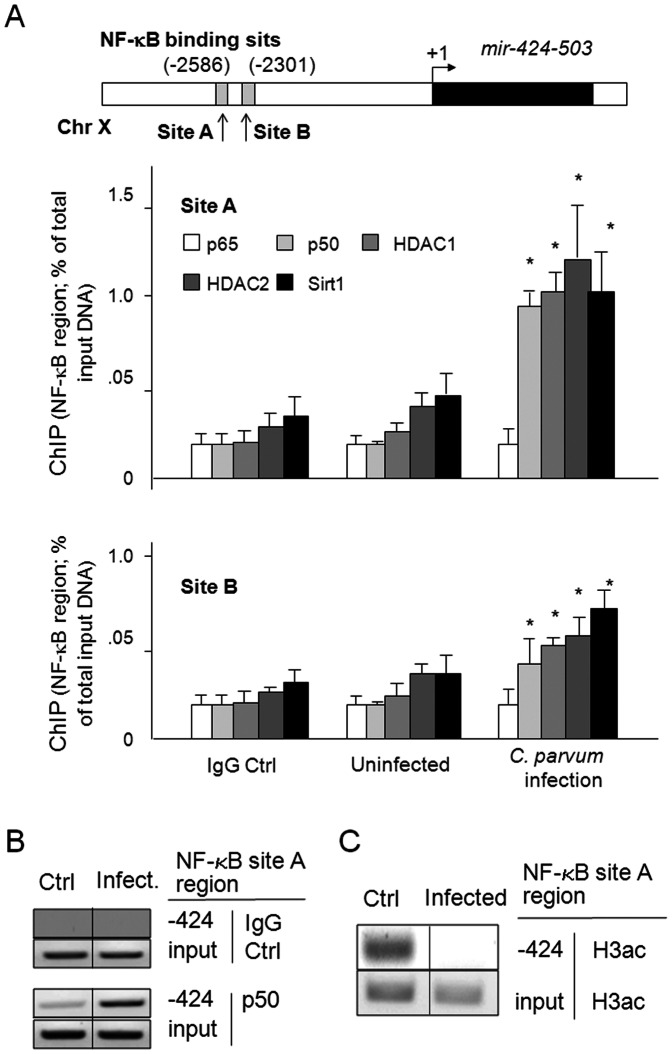
*C. parvum* infection increases recruitment of NF-kB p50 and HDAC complex and decreases H3 acetylation associated with the NF-kB promoter region of the *mir-424-503* gene promoter. **A**, Two potential binding sites for NF-kB are identified in the promoter of the *mir-424-503* gene. Increased promoter recruitment of p50, but not p65, and HDAC complex to the NF-kB associated site A region of *mir-424-503* gene was found in cells following *C. parvum* infection. H69 cells were exposed to *C. parvum* infection for 8 h. Promoter recruitment of p65, p50, HDAC1, HDAC2 and Sirt1 to the *mir-424-503* gene was assessed by ChIP analysis using primers covering the NF-kB-binding site A and site B regions within the promoter. The results were analyzed by real-time PCR and shown as the percentage of input. Data are averages of three independent experiments. The start of pri-miR-424-503 was indicated as +1. **B**, Representative PCR gels for p50 were shown as an example. **C**, Decrease of H3 acetylation in the NF-kB-associated region in the *mir-424-503* gene promoter in cells following *C. parvum* infection. H69 cells were exposed to *C. parvum* infection for 8 h. H3 acetylation associated with the NF-kB promoter site A region was assessed by ChIP analysis, using an antibody to H3Ac and primers covering the NF-kB-binding site A region within its promoter. *, p<0.05 ANOVA vs. the non-infected control.

We have identified two potential NF-kB binding sites within the promoter region of the *mir-424-503* gene (Fig. 5*A*). Increased promoter recruitment of p50, HDAC1, HDAC2 and Sirt1 to the NF-kB-associated site A region of the *mir-424-503* gene was found in cells following infection (Fig. 5*A and 5B*), as assessed by ChIP analysis using primers covering the NF-kB-binding region within the promoter. Moderate recruitment of p50, HDAC1, HDAC2 and Sirt1 to the NF-kB-associated site B region of *mir-424-503* gene was found in infected cells (Fig. 5*A*). No significant increase of promoter recruitment of p65 to both sites was detected in the infected cells (Fig. 5*A*). Because HDACs mediate gene expression through deacetylation of histones, such as H3 [4]-[6], we also measured the H3 acetylation associated with the NF-kB binding site A region of the *mir-424-503* gene promoter in infected cells. H3 acetylation associated with the NF-kB binding site A region of the *mir-424-503* gene promoter was obvious in the non-infected control cells (Fig. 5*C*). A significant decrease of H3 acetylation associated with this the promoter region was observed in the infected cells (Fig. 5*C*).

### *C. parvum* infection induces promoter recruitment of C/EBPβ associated with the NF-kB binding region of the mir-424-503 gene and increases direct interactions of C/EBPβ with p50 and Sirt1

**Figure 6:**
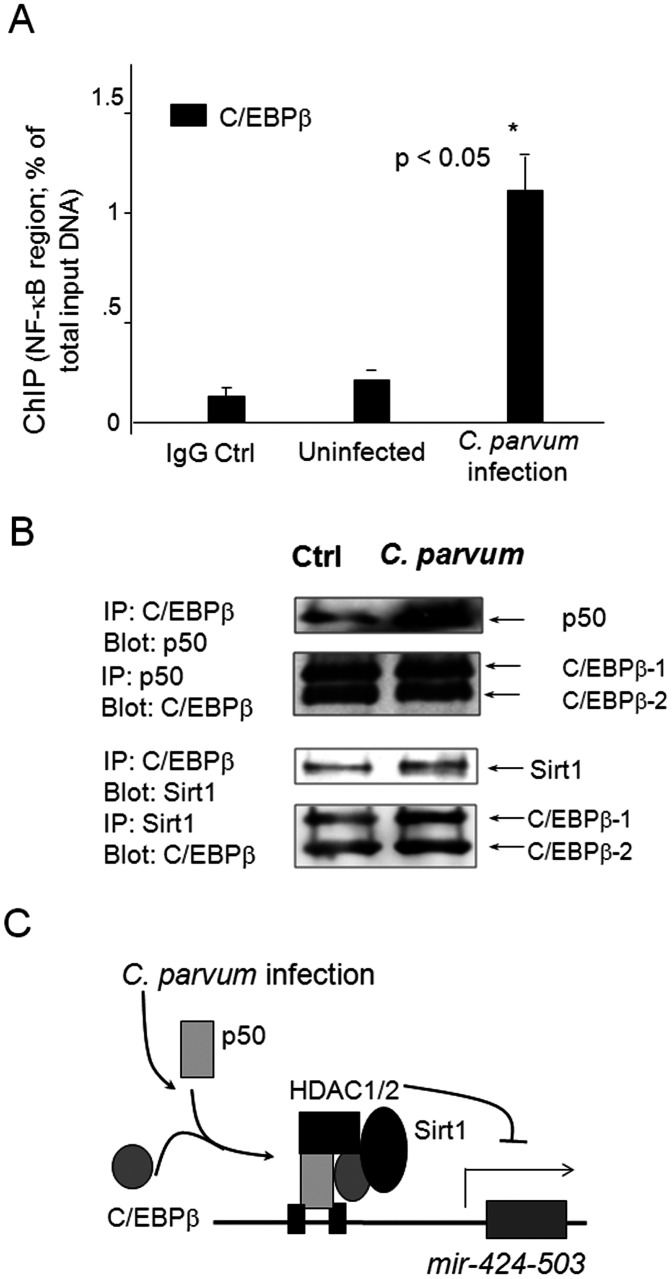
*C. parvum* infection increases recruitment of C/EBPβ associated with the NF-kB promoter region of the *mir-424-503* gene promoter. **A**, Increased promoter recruitment of C/EBPβ to the NF-kB-associated region of *mir-424-503* gene was found in cells following *C. parvum* infection. H69 cells were exposed to *C. parvum* infection for 8 h. Promoter recruitment of C/EBPβ to the *mir-424-503* gene was assessed by ChIP analysis using primers covering the NF-kB-binding region within the promoter. **B**, Interactions between p50 and C/EBPβ, as well as C/EBPβ and Sirt1, in cells following *C. parvum* infection as assessed by Co-IP. H69 cells were exposed to *C. parvum* infection for 8 h, followed by Co-IP analysis using antibodies to p50, C/EBPβ and Sirt1. C, The schematic of NF-kB-associated suppression of the *mir-424-503* gene in cells following *C. parvum* infection. Activation of NF-kB signaling promotes binding of p50 and C/EBPβ to the promoter region of the *mir-424-503* gene and increases recruitment of HDAC complex, resulting in trans-suppression of the *mir-424-503* gene in infected epithelial cells.

Given the fact that NF-kB p50 does not directly interact with Sirt1 [33], we speculated that promoter recruitment of Sirt1 may depend on the binding of other co-regulators or transcription factors. The transcription factor C/EBPβ can directly interact with both p50 and Sirt1 [27], [34], [35]. Thus, we tested the potential promoter recruitment of C/EBPβ associated with the NF-kB binding region of the *mir-424-503* gene in infected cells. Increased promoter recruitment was found in cells after infection (Fig. 6*A*), as assessed by ChIP analysis using primers covering the NF-kB-binding region within the promoter. To confirm the direct interactions between C/EBPβ and p50 or Sirt1, we performed co-IP analysis on the infected cells. A substantial portion of endogenous C/EBPβ and p50, as well as C/EBPβ and Sirt1, was co-immunoprecipitated together (Fig. 6*B*). This interaction is not due to DNA bridging, because it is resistant to ethidium bromide (data not shown). An increased interaction between C/EBPβ and p50, as well as C/EBPβ and Sirt1, was detected in cells after *C. parvum* infection (Fig. 6*B*), suggesting that *C. parvum* infection induces the formation of the p50-C/EBPβ-Sirt1/HDAC complex associated with the NF-kB binding region within the *mir-424-503* gene promoter, resulting in suppression of this gene (Fig. 6*C*).

### Upregulation of CX3CL1 in epithelial cells and increased mucosal infiltration of CX3CR1^+^ cells following *C. parvum* infection in the biliary tract in vivo

**Figure 7:**
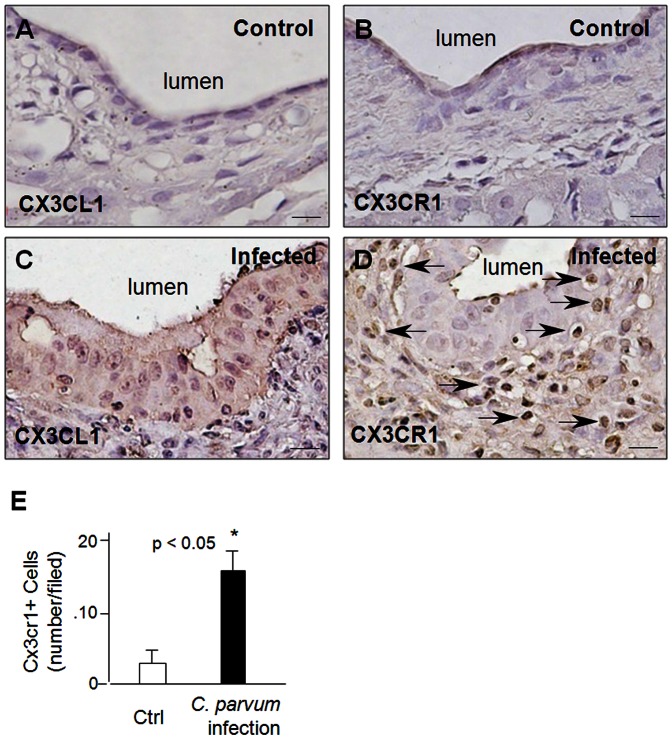
Upregulation of CX3CL1 in epithelial cells and an increase of mucosal infiltration of CX3CR1^+^ cells following *C. parvum* infection in the biliary tract in vivo. **A–D**, *C. parvum* oocysts were injected into the gallbladder of mice. Liver tissues at 2 weeks post-injection were collected and stained for CX3CL1 and CX3CR1. Upegulation of CX3CL1 was observed in epithelial cells of bile ducts from *C. parvum* infected animals (**A** and **C**). Increased infiltration of CX3CR1+ cells around the biliary tract was observed in the liver tissues from infected animals (**B** and **D**). Bar = 10 µm. **E**, quantification of CX3CR1^+^ cell infiltration around the biliary tract.

Membrane-bound CX3CL1 is known to function as an adhesion molecule to interact with immune cells that express CX3CR1, including CD4^+^ and CD8^+^ T-cells, NK cells, and monocytes [16]-[18]. Using a mouse model of biliary cryptosporidiosis via gallbladder injection of *C. parvum* oocysts [26], [28], we detected a significant increase in CX3CL1 protein level in biliary epithelial cells from mice infected with *C. parvum* for one week (Fig. 7*A and 7C*). In addition, increased infiltration of CX3CR1^+^ cells around the biliary tract was observed in liver tissues from infected animals (Fig. 7*B, 7D and 7E*).

## Discussion

Our findings reveal a novel role for acetylation of histones and NF-kB signaling in the regulation of chemokine CX3CL1 expression in epithelial cells upon *C. parvum* infection. *C. parvum* infection induces upregulation of CX3CL1 in biliary epithelial cells in vitro and increases biliary mucosal infiltration of CX3CR1^+^ cells during biliary infection in vivo. Release of miRNA-mediated post-transcriptional suppression through downregulation of miR-424 and miR-503 contributes to induction of CX3CL1, as evident by a modest increase in CX3CL1 mRNA but a robust induction of CX3CL1 protein in infected cells. Targeting of CX3CL1 by both miR-424 and miR-503 may reflect functional redundancy because they are transcribed from the same *mir-424-503* gene. Intriguingly, HDACs and NF-kB signaling regulate expression of CX3CL1 in epithelial cells to coordinate mucosal defense against *C. parvum* infection by suppressing the *mir-424-503* gene. Because induction of CX3CL1 and downregulation of miR-424 and miR-503 were also detected in epithelial cells in response to LPS stimulation, we speculated that HDACs and NF-kB signaling coordinate expression of CX3CL1 through suppressing the *mir-424-503* gene in epithelial cells upon microbial challenge in general.

*C. parvum* infection results in mucosal inflammation with infiltration of inflammatory cells, such as monocytes and neutrophils [11]–[15]. Our laboratory and others have previously shown that *C. parvum*-infected epithelial cells can participate in mucosal inflammation by activating NF-kB signaling to produce adhesion molecules and C-X-C chemokines, such as ICAM1, IL-6, IL-8 and Gro-α [12]–[15], [36]–[38]. Proinflammatory cytokines like IL-1β and TNF-α, produced by many different cell types, can induce and amplify the secretion of various chemokines and therefore promote the recruitment of inflammatory cells in the mucosa [37], [38]. Our results extend those findings by demonstrating that CX3CL1 was also produced in response to *C. parvum* infection. Increased infiltration of CX3CR1^+^ cells was observed along the biliary tract during *C. parvum* biliary infection in vivo. We hypothesize that along with other adhesion molecules and cytokines/chemokines, CX3CL1 expression and release from epithelial cells also contributes to the mucosal anti-*C. parvum* defense through recruitment of immune cells into the mucosa; nevertheless, the involvement of CX3CL1 in this mechanism remains to be demonstrated in vivo using neutralizing antibodies or epithelial specific CX3CL1 KO mice.

Among miRNAs suppressed in host epithelial cells following *C. parvum* infection, we identified that miR-424 and miR-503 target 3′UTR of CX3CL1 mRNA, contributing to induction of CX3CL1 in infected epithelial cells. Similar to other RNA molecules, most miRNAs are initially transcribed as primary transcripts (termed pre-miRNAs) by Poly II and processed by the RNase III Drosha (in the nucleus) and a second RNase III Dicer (in the cytoplasm) to generate mature miRNA molecules [39], [40]. The *mir-424-503* gene locus at chromosome X codes the mature form of both miR-424 and miR-503 [32]. Our previous work demonstrated that promoter binding of NF-kB p65 complex triggers transactivation of miRNA genes in infected cells [21]–[23]. In contrast, promoter binding of NF-kB p50:p50 repressor complex suppresses transcription of *let-7i* miRNA gene [24], [27]. In the current study, our results indicate that transcriptional suppression of the *mir-424-503* gene is associated with an increased promoter recruitment of NF-kB p50. Intriguingly, promoter recruitment of several HDACs, including HDAC1, HDAC2 and Sirt1, was also detected in infected cells. Such promoter recruitment of NF-kB p50 and HDAC complex appears to be associated with a decrease of H3 acetylation and account for the repression of the *mir-424-503* gene in infected cells. Moreover, promoter recruitment of C/EBPβ to the *mir-424-503* gene is also increased in *C. parvum*-infected cells. Given the fact that NF-kB p50 can directly interact with C/EBPβ [34] but not Sirt1 [33], it is possible that promoter recruitment of Sirt1 may depend on binding of C/EBPβ. Indeed, a substantial portion of endogenous C/EBPβ and Sirt1 co-immunoprecipitated together, and increased interactions of C/EBPβ with NF-kB p50 and Sirt1 were detected in infected cells.

Evidence has accumulated showing that HDACs have immunomodulatory activity and are important to regulation of host anti-microbial defense [41]. Multiple reports have shown that HDAC inhibitors possess suppressive effects on immune response gene induction [7], [41]. Individual cytokines that are induced by microbial components triggering TLRs were reported to be inhibited by HDAC inhibitors [7], [8]. Conversely, treatment of mice with the HDAC inhibitors increased their susceptibility to pneumonia by *Klebsiella pneumonia* as well as systemic candidiasis [7]. HDAC inhibition conferred protection in models of septic shock by limiting the cytokine burst [41]. It has been reported previously that patients treated with HDAC inhibitors show an increased susceptibility to develop severe infection even without neutropenia [42]. However, the extent of HDAC immunomodulatory effects and possible functional consequences during infections are largely unknown [41]. Given the fact that HDAC inhibitors have become promising candidates for the treatment of different types of cancer, a better understanding of the mechanisms involved in HDAC-mediated immunomodulatory activity is of critical clinical significance. Our finding of an HDAC-associated suppression of the *mir-424-503* gene, and possible induction of epithelial expression of CX3CL1 upon microbial challenge, provides new insights into the molecular mechanisms of HDAC immunomodulatory functions.

In summary, we demonstrated that CX3CL1 is upregulated in biliary epithelial cells upon microbial challenge. Induction of CX3CL1 may be associated with downregulation of miR-424 and miR-503, both of which target the CX3CL1 3’UTR, suppress its translation and induce RNA degradation. Our findings indicate that HDACs and NF-kB signaling coordinate downregulation of the *mir-424-503* gene to promote mucosal defense through modulating CX3CL1 expression in epithelial cells.

## Supporting Information

Figure S1 **Upregulation of CX3CL1 in epithelial cells in response to LPS stimulation.** H69 cells were exposed to LPS for up to 24 h, followed by Western blot (**A**) and qRT-PCR (**B**) analysis for CX3CL1. Representative Western blots were shown and β-actin was blotted as the protein loading control. GAPDH mRNA was used to normalize the CX3CL1 mRNA levels. Data are averages of three independent experiments. *, p<0.05 ANOVA vs. the non-treated cells.
(TIF)

Figure S2 **HDAC- and Dicer-dependent expression of CX3CL1 in epithelial cells in response to LPS stimulation.** **A,** Treatment of cells with HDAC inhibitors, TSA and EX527, and NF-kB inhibitor SC514 attenuated LPS-induced upregulation of CX3CL1. H69 cells were exposed to LPS for up to 24 h, followed by qRT-PCR analysis for CX3CL1. **B,** Knockdown of Dicer blocked the inhibitory effects of TSA on LPS-induced CX3CL1 expression. Cells were treated with the siRNA to Dicer and then exposed to LPS for 12 h in the absence or presence of TSA, followed by qRT-PCR analysis for CX3CL1. Data are averages of three independent experiments. *, p<0.05 ANOVA vs. the non-LPS control (in **A**); ^#^, p<0.05 ANOVA vs. LPS-stimulated cells (in A) or non-TSA-treated cells (as indicated in **B**).
(TIF)

Figure S3 **Downregulation of miR-424 and miR-503 in epithelial cells in response to LPS stimulation.** H69 cells were exposed to LPS for 8h, followed by microarray. Expression levels of miR-424 and miR-503 by microarray are presented as the log_2_ (Hy5/Hy3) ratios.
(TIF)
