# Supplementary figures and images for: Histone Deacetylases and NF-kB Signaling Coordinate Expression of CX3CL1 in Epithelial Cells in Response to Microbial Challenge by Suppressing miR-424 and miR-503
Source: PLoS One. 2013 May 28;8(5):e65153. doi: 10.1371/journal.pone.0065153 (PMC3665534; doi:10.1371/journal.pone.0065153)

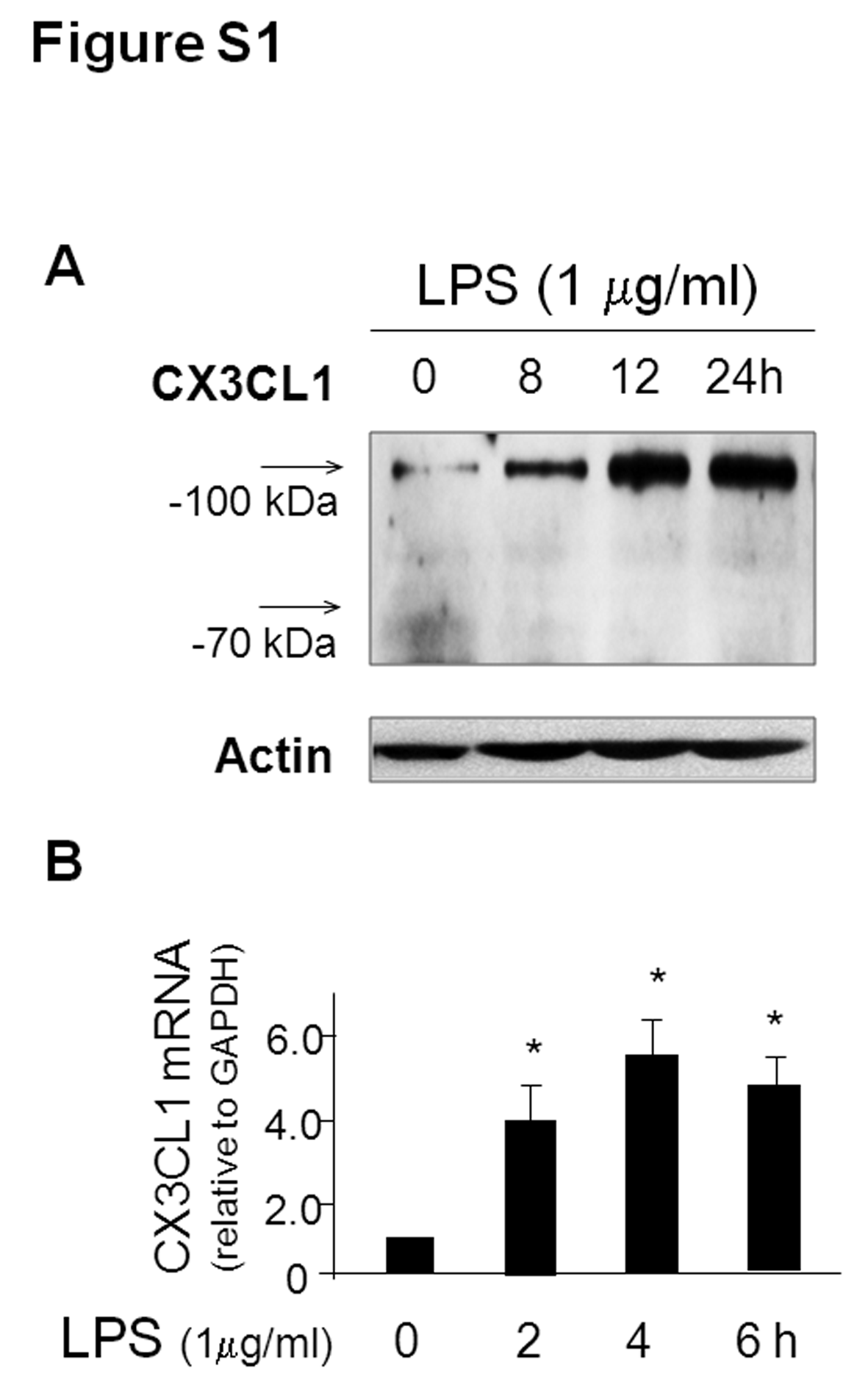

Supplement: Figure S1 — Upregulation of CX3CL1 in epithelial cells in response to LPS stimulation. H69 cells were exposed to LPS for up to 24 h, followed by Western blot (A) and qRT-PCR (B) analysis for CX3CL1. Representative Western blots were shown and β-actin was blotted as the protein loading control. GAPDH mRNA was used to normalize the CX3CL1 mRNA levels. Data are averages of three independent experiments. *, p<0.05 ANOVA vs. the non-treated cells. (TIF) [file pone.0065153.s001.tif]

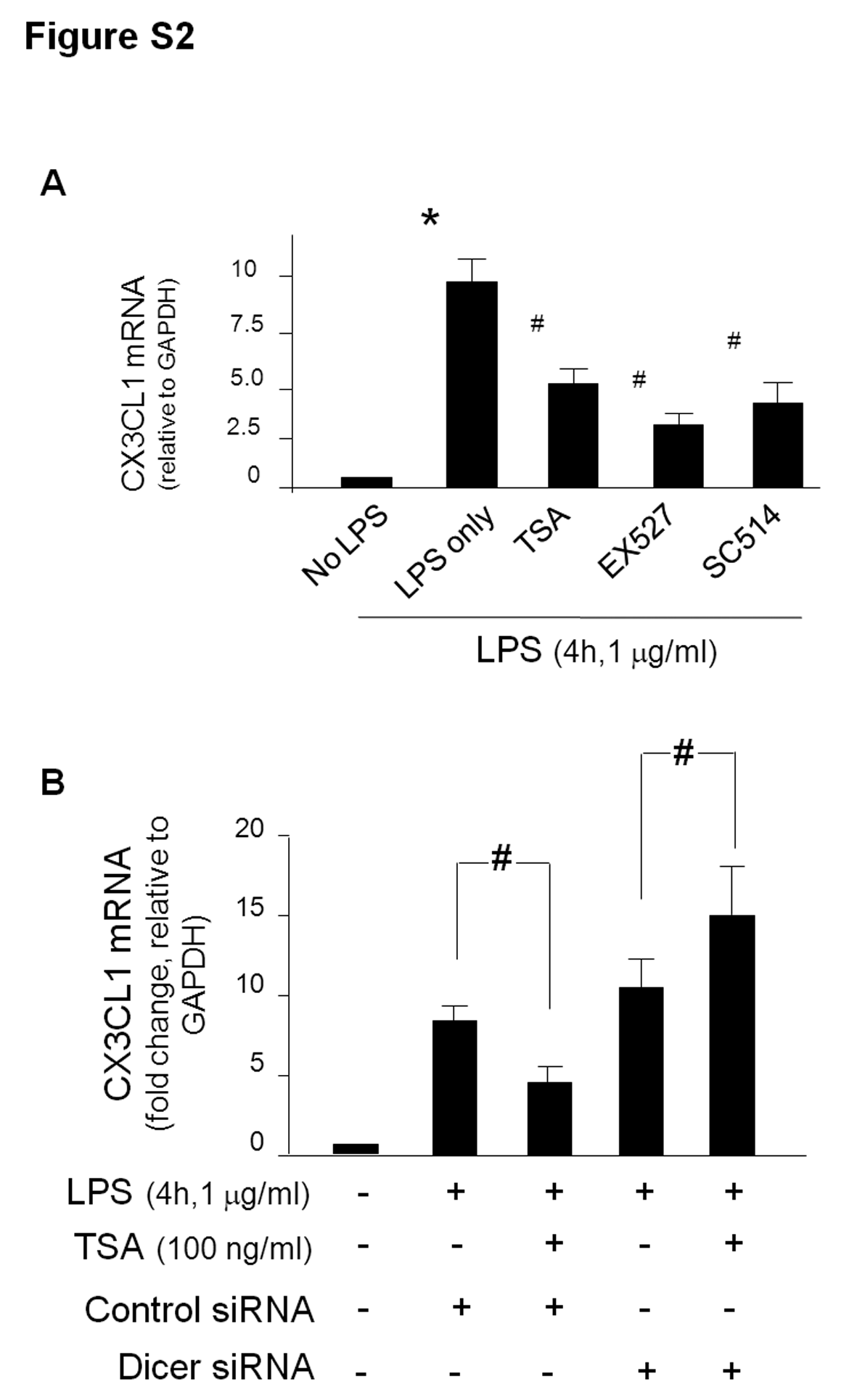

Supplement: Figure S2 — HDAC- and Dicer-dependent expression of CX3CL1 in epithelial cells in response to LPS stimulation. A, Treatment of cells with HDAC inhibitors, TSA and EX527, and NF-kB inhibitor SC514 attenuated LPS-induced upregulation of CX3CL1. H69 cells were exposed to LPS for up to 24 h, followed by qRT-PCR analysis for CX3CL1. B, Knockdown of Dicer blocked the inhibitory effects of TSA on LPS-induced CX3CL1 expression. Cells were treated with the siRNA to Dicer and then exposed to LPS for 12 h in the absence or presence of TSA, followed by qRT-PCR analysis for CX3CL1. Data are averages of three independent experiments. *, p<0.05 ANOVA vs. the non-LPS control (in A); #, p<0.05 ANOVA vs. LPS-stimulated cells (in A) or non-TSA-treated cells (as indicated in B). (TIF) [file pone.0065153.s002.tif]

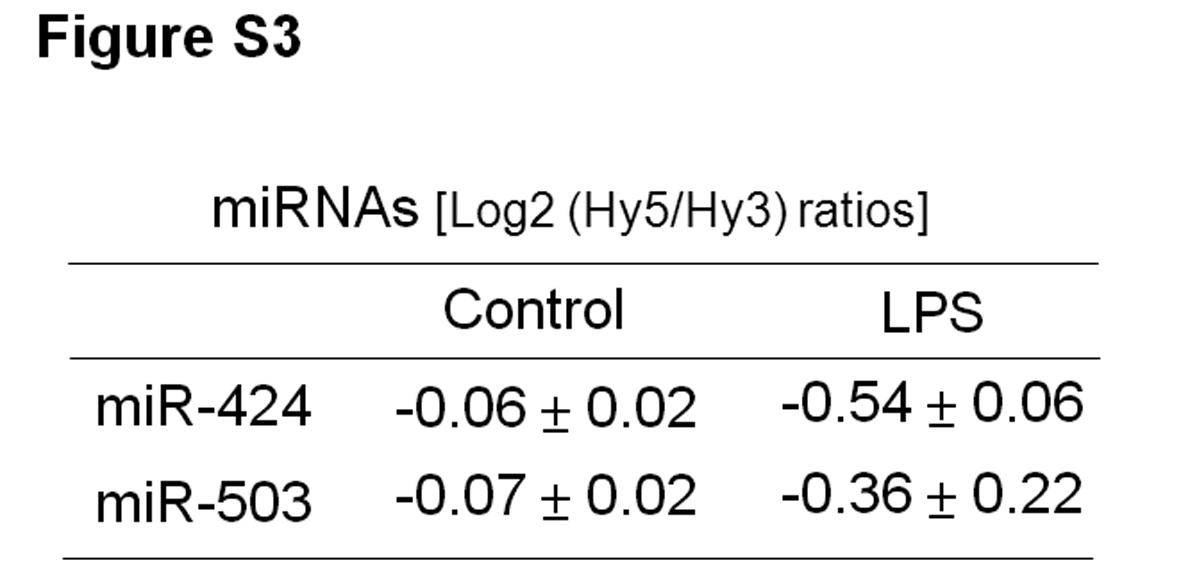

Supplement: Figure S3 — Downregulation of miR-424 and miR-503 in epithelial cells in response to LPS stimulation. H69 cells were exposed to LPS for 8h, followed by microarray. Expression levels of miR-424 and miR-503 by microarray are presented as the log2 (Hy5/Hy3) ratios. (TIF) [file pone.0065153.s003.tif]
